# Improved Gate Dielectric Deposition and Enhanced Electrical Stability for Single-Layer MoS_2_ MOSFET with an AlN Interfacial Layer

**DOI:** 10.1038/srep27676

**Published:** 2016-06-09

**Authors:** Qingkai Qian, Baikui Li, Mengyuan Hua, Zhaofu Zhang, Feifei Lan, Yongkuan Xu, Ruyue Yan, Kevin J. Chen

**Affiliations:** 1 Department of Electronic and Computer Engineering, Hong Kong University of Science and Technology, Clear Water Bay, Hong Kong SAR, China; 2 The 46th Research Institute, CETC, Tianjin 300220, China

## Abstract

Transistors based on MoS_2_ and other TMDs have been widely studied. The dangling-bond free surface of MoS_2_ has made the deposition of high-quality high-k dielectrics on MoS_2_ a challenge. The resulted transistors often suffer from the threshold voltage instability induced by the high density traps near MoS_2_/dielectric interface or inside the gate dielectric, which is detrimental for the practical applications of MoS_2_ metal-oxide-semiconductor field-effect transistor (MOSFET). In this work, by using AlN deposited by plasma enhanced atomic layer deposition (PEALD) as an interfacial layer, top-gate dielectrics as thin as 6 nm for single-layer MoS_2_ transistors are demonstrated. The AlN interfacial layer not only promotes the conformal deposition of high-quality Al_2_O_3_ on the dangling-bond free MoS_2_, but also greatly enhances the electrical stability of the MoS_2_ transistors. Very small hysteresis (Δ*V*_th_) is observed even at large gate biases and high temperatures. The transistor also exhibits a low level of flicker noise, which clearly originates from the Hooge mobility fluctuation instead of the carrier number fluctuation. The observed superior electrical stability of MoS_2_ transistor is attributed to the low border trap density of the AlN interfacial layer, as well as the small gate leakage and high dielectric strength of AlN/Al_2_O_3_ dielectric stack.

Molybdenum disulfide (MoS_2_), as a layered material from the transition metal dichalcogenide (TMD) family, has been widely studied in recent years^1^,^2^,^3^, for its intriguing properties such as atomic-layer thickness, tunable bandgap^4^, high mobility^3^ and good thermal stability^1^. MoS_2_ MOSFET has been shown to exhibit suppressed short channel effect and has the potential to be used in the next generation nanoelectronics^2^,^5^. Many other applications based on single- or multi-layer MoS_2_, such as flexible electronics^6^,^7^,^8^, photon detectors^9^,^10^ and gas sensors^11^, have been demonstrated. Single-layer MoS_2_ also offers new opportunities in novel piezoelectronics^12^,^13^ and valleytronics^14^,^15^. Moreover, the recent advance in wafer-scale deposition of high-quality MoS_2_ films^16^ has made these MoS_2_ based applications even more promising.

However, the performance of MoS_2_ MOSFET is very sensitive to the ambient conditions and electrical stress^17^,^18^,^19^. As reported previously, when exposed to air, the MoS_2_ MOSFETs could exhibit large performance variation in terms of large shift in threshold voltage (i.e. hysteresis), due to the water or oxygen adsorbates^18^,^19^. This threshold voltage instability (Δ*V*_th_) is detrimental to both logic and analog circuit applications. Moreover, these ambient adsorbates will further degrade the carrier mobility and cause severe current fluctuation noise^20^. The MoS_2_ MOSFET has to be passivated in order to alleviate the ambient influence and achieve reliable performance. Because of the dangling-bond-free nature of MoS_2_ surface, the deposition of high quality dielectric on MoS_2_ can be challenging^21^,^22^,^23^,^24^,^25^. Even with passivation, MoS_2_ MOSFETs still exhibit significant hysteresis problem^7^,^26^, due to high-density traps at MoS_2_/dielectric interface or in the gate dielectric.

Both hexagonal boron nitride (hBN) and aluminum nitride (AlN) are suggested to be ideal gate dielectrics for MoS_2_ FETs^27^. It has already been demonstrated that the hysteresis of MoS_2_ transistor was greatly reduced by using the exfoliated hBN as gate dielectric, benefiting from the good MoS_2_/dielectric interface^8^. But as a layered material, hBN is usually obtained by mechanical exfoliation^8^,^28^ or synthesized at high temperatures^29^,^30^, which makes it difficult to be deposited reliably on large scale as top-gate dielectric. In contrast to the layered hBN, AlN is a bulk material with a larger bandgap of 6.3 eV and higher dielectric constant of 9.14^27^,^31^, and can be deposited uniformly on large samples by PEALD^32^ or thermal ALD^33^. Moreover, our recent result has shown that by using AlN and Al_2_O_3_ stack as gate dielectric, AlN is capable of isolating the channel from the bulk traps of Al_2_O_3_ and achieving a low border trap density^34^,^35^.

In this paper, we report the experimental demonstration of single-layer MoS_2_ MOSFETs with AlN/Al_2_O_3_ as top-gate dielectric. By the insertion of AlN interfacial layer, the gate dielectrics scaling down to as thin as 6 nm are realized. The electrical stabilities of the fabricated devices are systematically characterized under different conditions. The transistor shows very small hysteresis even under large gate biases and high temperatures. Low-frequency noise characterization is conducted and the MoS_2_ transistor exhibits suppressed current fluctuation. The observed excellent threshold voltage stability is contributed by the low border trap density of AlN near the MoS_2_ interface, as well as the small gate leakage and high dielectric strength of AlN/Al_2_O_3_.

## Results and Discussion

MoS_2_ samples used here were synthesized on sapphire substrate by CVD method using high purity MoO_3_ and S powder as precursors. The triangular MoS_2_ flakes have a size of about 100 μm (See Supplementary Figure S1). The Raman characterization shows peak distance of 19 cm^−1^ for 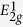 and *A*_1*g*_, suggesting the MoS_2_ flakes are single layered^36^. The MoS_2_ flakes were then transferred to a silicon substrate capped with 300-nm thermally grown SiO_2_. Depositing dielectric on the dangling-bond-free MoS_2_ is a challenging task^21^,^22^,^23^,^24^,^25^. At first, the process for depositing AlN/Al_2_O_3_ dielectric stack on MoS_2_ was carefully tested. Both AlN and Al_2_O_3_ were deposited using the Oxford Instruments OpAL ALD system. AlN was deposited at 170 °C by using trimethylaluminum (TMA) and remote N_2_ plasma (20 sccm with a coil power of 25 W) as Al and N sources. The growth temperature and the RF power were optimized to minimize the plasma damage to the MoS_2_, resulting in a relatively slow growth rate of 0.18 Å/cycle. The detailed optimization processes are provided in Supplementary Figure S2. After the deposition of 2-nm AlN, 18-nm Al_2_O_3_ was grown *in-situ* under thermal ALD mode at 200 °C by using TMA and water vapor as Al and O sources.

**Figure 1:**
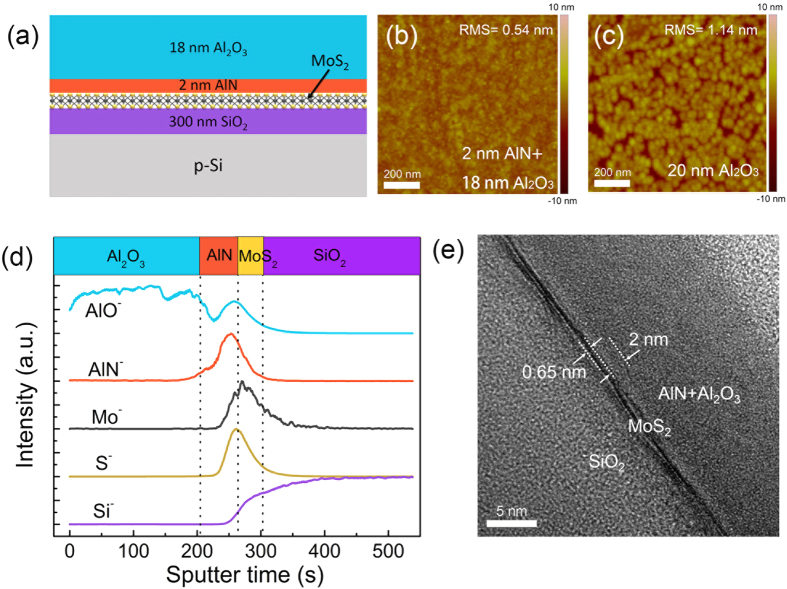
(**a**) Schematic of MoS_2_ sample after the deposition of AlN/Al_2_O_3_. (**b**) AFM image of MoS_2_ surface after the deposition of 2-nm AlN and 18-nm Al_2_O_3_. (**c**) AFM image of MoS_2_ surface after the direct deposition of 20-nm Al_2_O_3_. (**d**) Element distribution profiles from ToF-SIMS measurement and (**e**) Cross-sectional HRTEM image of the structure in (**a**).

The schematic structure of the single-layer MoS_2_ sample after the deposition of 2-nm AlN and 18-nm Al_2_O_3_ is shown in Fig. 1a. The surface morphology was characterized by AFM and shown in Fig. 1b. Continuous and smooth surface was obtained. Within a 1 μm^2^ area, the root mean square (RMS) surface roughness is 0.54 nm. In our experiment, the bare SiO_2_/Si wafer usually has a surface roughness of 0.15 nm. However, since the MoS_2_ samples are transferred to the SiO_2_/Si wafer, the surface roughness increases to 0.35–0.5 nm and dominates the above measured results. In contrast, for the MoS_2_ sample covered by 20-nm Al_2_O_3_ deposited at 200 °C, as shown by the AFM image in Fig. 1c, the dielectric film is full of broken areas due to weak adhesion of the precursors (TMA and water) on the dangling-bond-free MoS_2_ surface, which is consistent with previous reports^23^,^25^. Since it has been suggested that organic or solvent residue can assist the successful deposition of dielectrics on MoS_2_^22^, the failure of direct deposition of Al_2_O_3_ on the MoS_2_ sample also reflects that the MoS_2_ has maintained a clean surface during the fabrication process. The successful deposition of AlN/Al_2_O_3_ on MoS_2_ could be the result of the relatively low growth temperature of AlN. However, we found that even when the ALD growth temperature of Al_2_O_3_ was reduced to 170 °C, similar poor quality was still observed when Al_2_O_3_ was directly deposited on MoS_2_ (See Supplementary Figure S1). Thus, we conclude that the improved surface morphology and quality of AlN/Al_2_O_3_ dielectric stack on the dangle-bond-free MoS_2_ surface is mainly the benefits of the low-power remote nitrogen plasma during the PEALD growth of AlN, which is similar to the O_2_ plasma functionalization of the multilayer MoS_2_ that was used to promote the ALD deposition of Al_2_O_3_^25^. Even though the remote pure N_2_ plasma is very mild to single-layer MoS_2_ during several hours’ treatment (See Supplementary Figure S4), defects are still observed due to the possible Ar/H plasma damage during dose and purge of TMA (See Supplementary Figure S2(c)). At present, it cannot be determined to what extent the plasma damage facilitates the uniform dielectric deposition. Further experiments are needed to verify the possibility of AlN deposition on the dangling-bond free MoS_2_ only by the physical absorption of N-ion on the MoS_2_ surface, even for single-layer MoS_2_.

The successful deposition of AlN/Al_2_O_3_ stack on MoS_2_ was further verified by Time-of-Flight Second Ion Mass Spectrometry (ToF-SIMS), as shown in Fig. 1d. In this plot each relevant element’s intensity has been normalized by its peak value and offset vertically for clearer view. We can clearly identify the peak for AlN^−^, which appears ahead of Mo^−^ and S^−^ during the surface sputtering. Figure 1e shows the cross-sectional high resolution transmission electron microscopic (HRTEM) image of the SiO_2_/MoS_2_/AlN/Al_2_O_3_ structure. The single-layer MoS_2_ can be clearly identified. Both AlN and Al_2_O_3_ are in amorphous state without a visible distinct junction boundary between them. The N atom concentration is more likely to be higher near the MoS_2_ interface, due to the possible oxidation of AlN surface during the subsequent Al_2_O_3_ growth. The relatively bright area near MoS_2_/AlN interface might be the result of smaller density of AlN compared to that of Al_2_O_3_, which causes less electron scattering in the bright field TEM and thus becomes brighter. The AlN/Al_2_O_3_ layers are uniformly grown on MoS_2_ surface with no gaps or agglomerates, implying a reliable deposition process of AlN/Al_2_O_3_ as gate dielectric for MoS_2_ transistors.

**Figure 2:**
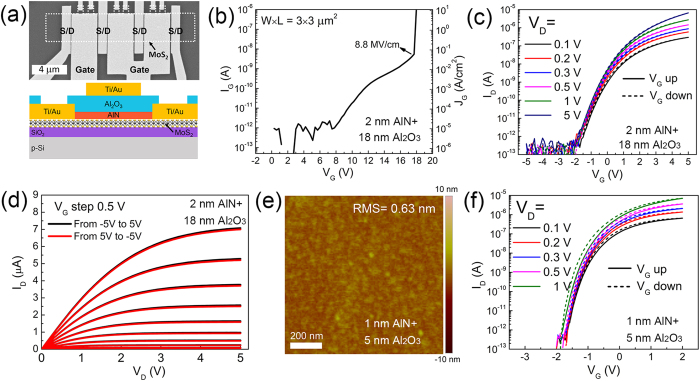
(**a**) SEM image and schematic of single-layer MoS_2_ transistor with AlN/Al_2_O_3_ as top-gate dielectric. (**b**) Forward gate *I-V* characteristics with gate dielectric breakdown. (**c**) Transfer curves (*I*_D_ vs. *V*_G_) at different drain biases with the gate voltage swept from −5 V to 5 V (solid lines, up-sweep) then back to −5 V (dashed line, down-sweep). (**d**) Output curves by stepping *V*_G_ up (black) and down (red) with a *V*_G_ step 0.5 V. Performances of (**b–d**) are measured for transistor with 2-nm AlN/18-nm Al_2_O_3_ dielectric stack. (**e**) AFM image of MoS_2_ surface after the deposition of 1-nm AlN and 5-nm Al_2_O_3_. (**f**) Transfer curves swept from −2 V to 2 V then back to −2 V for transistor with 1-nm AlN and 5-nm Al_2_O_3_ as gate dielectric.

Single-layer MoS_2_ transistors with 2-nm AlN/18-nm Al_2_O_3_ as top-gate dielectric were fabricated. Source/drain contacts were defined by electron-beam photolithography (EBL), followed by e-beam evaporation of 10-nm Ti and 50-nm Au and lift-off. The MoS_2_ flakes are further patterned into the designed channel size by EBL and O_2_ plasma etch. Then an AlN/ Al_2_O_3_ (2 nm/18 nm) stack was deposited on top of MoS_2_ as gate dielectric following the afore-described PEALD/ALD procedure. Another EBL was conducted to define the top gate, followed by e-beam evaporation of Ti/Au (10 nm/50 nm) and lift-off. Finally, the contact holes were formed by etching AlN/Al_2_O_3_ with developer FHD-5. The SEM image of fabricated devices is shown in Fig. 2a, together with the schematic cross-sectional view of the device. The channel width and gate length are 3 μm. There is 100-nm spacing between the gate and source/drain contacts. The MoS_2_ transistors were measured at room temperature in atmosphere by Agilent B1505A device analyzer/curve tracer. Figure 2b shows result of the gate leakage and the hard breakdown test for the gate dielectric. The current of about 1 pA when biased at small gate voltage is mainly limited by the equipment resolution. The AlN/Al_2_O_3_ dielectric shows a small leakage of about 0.1 pA/μm^2^ for gate electric field as high as 4 MV/cm. At the same time a high breakdown electric field of 8.8 MV/cm is measured.

Figure 2c shows the transfer curves measured under different drain voltage biases ranging from 0.1 V to 5 V for transistor with 2-nm AlN and 18-nm Al_2_O_3_ as gate dielectric. During the measurements the gate voltage is swept from −5 V to 5 V then back to −5 V again. The transfer curves for sweeping *V*_G_ up and *V*_G_ down are plotted by the solid and dashed lines respectively. Remarkably the transfer curves exhibit very small hysteresis for all the drain voltage biases. A large on/off ratio of about 10^6^ is achieved, and the off-current is still limited by the equipment current resolution. The field effect mobility is extracted to be 3.3 cm^2^/V · s by using equation μ = *dI*_*D*_/*dV* × *L*/(*WC*_*ox*_*V*_*D*_) in four-probe measurement configuration, during which *ε* = 9 is adopted as the relative dielectric constant for both AlN and Al_2_O_3_ to calculate *C*_*ox*_. This observed mobility is comparable to previous results for single-layer MoS_2_ transistors with MoS_2_ channel exposed to air^8^,^37^, but is smaller than that of 13–16 cm^2^/V · s for similar devices passivated by ALD Al_2_O_3_^38^,^39^. The relatively smaller mobility might be the results of varied CVD growth and fabrication conditions^40^ or specifically the remote plasma damage introduced by the PEALD growth of AlN in our case (See Supplementary Figure S4). Because single-layer MoS_2_ has only one atomic layer thickness and lacks the Thomas-Fermi screening effect to mitigate the impacts from the MoS_2_/dielectric interfaces^3^, the mobility of single-layer MoS_2_ becomes more sensitive to the dielectric environments and the plasma damage, and is reported to be smaller than that of multilayer ones^8^,^37^,^41^. By using high-quality exfoliated multilayer MoS_2_ in the future, which is less vulnerable to potential variations^37^ and more resistant to surface plasma treatment^25^,^42^, higher mobility can be achieved. Figure 2d shows the output curves, the black and red curves represent the results for stepping *V*_G_ up and *V*_G_ down respectively. Very good current saturation is observed, and the *I*_D_-*V*_D_ near *V*_*D*_ = 0 *V* exhibits linear relationship. There is almost no discrepancy between the output curves for sweeping *V*_G_ up and *V*_G_ down, suggesting reliable performance.

To fully exploit the potential of the AlN interfacial layer in promoting the uniform deposition of high-quality dielectric on the dangling-bond free MoS_2_ surface, gate dielectric consisting of 1-nm AlN and 5-nm Al_2_O_3_ is deposited, which is the thinnest dielectric ever reported for MoS_2_ transistor^25^. The MoS_2_ surface after the dielectric deposition is characterized by AFM and shown in Fig. 2e. Even with greatly reduced thickness, the AlN/Al_2_O_3_ on the dangling-bond free MoS_2_ still maintains a smooth surface with no pinholes, implying uniform nucleation sites provided by the 1-nm AlN interfacial layer. Figure 2f shows the transfer curves for MoS_2_ transistor with this ultra-thin stack as gate dielectric. Effective gate modulation and small hysteresis are observed, suggesting that by using PEALD AlN as an interfacial layer, high-quality dielectric even with sub-10 nm thickness can be successfully deposited on the dangling-bond free MoS_2_, which is very important for the continuous scaling down of MoS_2_ FET for higher performance.

**Figure 3:**
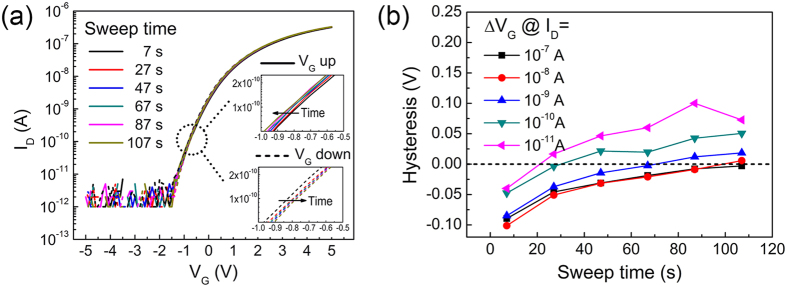
(**a**) Transfer curves with different sweep time for transistor with 2-nm AlN/18-nm Al_2_O_3_ gate dielectric. The *V*_G_ is swept from −5 V to 5 V then back to −5 V again, and *V*_D_ is 0.1 V. The two insets show the zoomed-in transfer curves for sweeping *V*_G_ up and *V*_G_ down respectively. (**b**) Hysteresis extracted from (**a**) by calculating the difference of *V*_G_ corresponding to a specific *I*_D_ during sweeping *V*_G_ down and *V*_G_ up. Different *I*_D_ criteria are used.

The transfer curves for transistor with 2-nm AlN/18-nm Al_2_O_3_ gate dielectric shown in Fig. 2c were measured with a total sweep time of 7 s. However there are reports suggesting that the threshold voltage instability can be strongly affected by the sweep rate^18^,^19^. So we also measured the transfer curves with different sweep time ranging from 7 s to 107 s as shown in Fig. 3a. Still no obvious hysteresis is observed. By zooming in the transfer curves as shown in the two insets of Fig. 3a, we can identify the trends of shifting in the transfer curves. With increased sweep time, the transfer curve continuously shifts left during the up-sweep, and shifts right during the down-sweep. The above observation suggests that more trapped electrons are emitted with a slower up-sweep, and more electrons are trapped with a slower down-sweep. These threshold voltage shift trends result in an increased hysteresis as plotted in Fig. 3b, in which the hysteresis is calculated by the difference of *V*_G_ corresponding to the same *I*_D_ during the up- and down-sweep. Different *I*_D_ criteria have been used, and the resultant hysteresis differs from each other due to the complex trapping and stabilization processes involved during a complete sweep. We find that smaller *I*_D_ criterion yields larger hysteresis. Interestingly, a negative hysteresis is observed when the sweep time is short, which might be the result of gate-side electron injection^43^ and is consistent with the pulsed I-V measurement results (See Supplementary Figure S5). In general, with increased sweep time, the hysteresis increases but tends to be stabilized and is limited within 0.1 V for all the *I*_D_ criteria. Such a small hysteresis is the best result ever reported to date for MoS_2_ FET.

**Figure 4:**
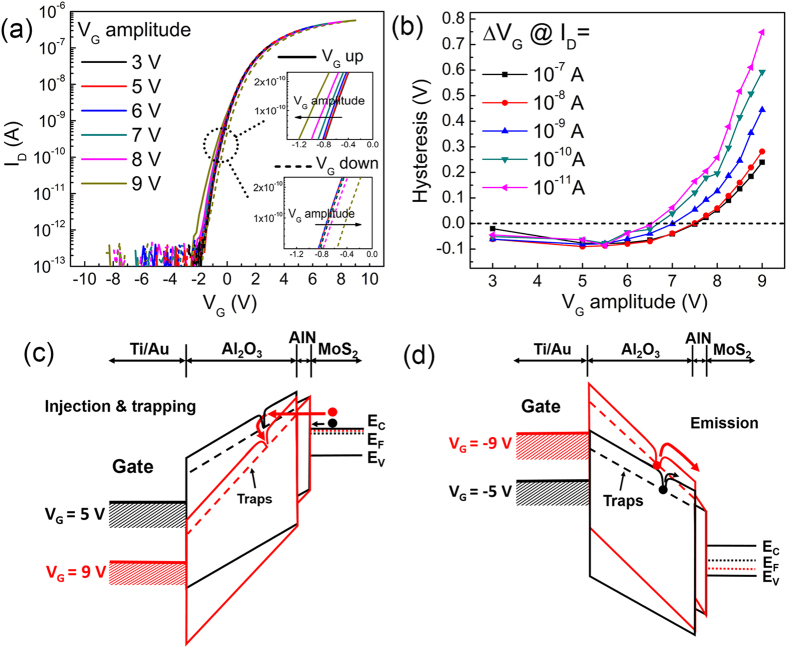
(**a**) Transfer curves measured with different *V*_G_ sweep amplitude for transistor with 2-nm AlN/18-nm Al_2_O_3_ gate dielectric. The sweep time is 7 s and the drain bias is 0.1 V. The two insets show the zoomed-in transfer curves during the up- and down-sweep. (**b**) Hysteresis extracted from (**a**). (**c**) Schematic band diagram when the gate are positively biased at 5 V and 9 V. With a larger sweep amplitude, enhanced electron injection and trapping would occur since the tunneling barrier is reduced. (**d**) Schematic band diagram when the gate is negatively biased at −5 V and −9 V. The detrapping of electrons is assisted by higher electric field.

To better understand the observed hysteresis, transfer curves with different *V*_G_ sweep amplitudes for transistor with 2-nm AlN/18-nm Al_2_O_3_ gate dielectric were measured and plotted in Fig. 4a. The gate is swept from negative to positive then back to negative voltage with a total sweep time of 7 s. The hysteresis becomes more pronounced with increased *V*_G_ amplitude. The hysteresis is extracted quantitatively as shown in Fig. 4b. It is observed that the hysteresis has little change when *V*_G_ amplitude is smaller than 7 V, but increases more significantly when *V*_G_ amplitude is larger. It is also noticed that there is almost no *V*_th_ shift until the *V*_G_ amplitudes is larger than 8 V during the down-sweep. Recalling that the gate leakage in Fig. 2b also becomes prominent only when *V*_G_ is larger than 8 V, this *V*_th_ shift can then be correlated with the injection and trapping of electrons inside the gate dielectric^44^,^45^ as schematically shown in Fig. 4c. On the other hand, when the gate is negatively biased, the high electric field will enhance the emission of electrons trapped inside the gate dielectric, accounting for the negative *V*_th_ shift at larger *V*_G_ amplitude in the down-sweep, as schematically shown in Fig. 4d. The above observation reveals that the MoS_2_ transistor with AlN/Al_2_O_3_ as top-gate dielectric presents small hysteresis even under large gate voltage bias, and the observed weak threshold voltage instability is mainly caused by the gate leakage at high gate electric field and the resultant trapping/detrapping of electrons in the border traps (within the bulk Al_2_O_3_ but close to the dielectric/MoS_2_ interface).

**Figure 5:**
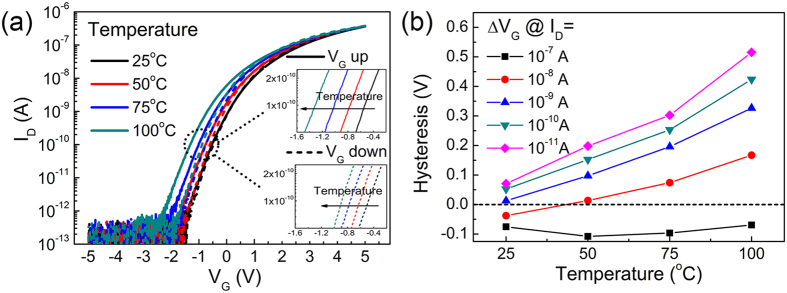
(**a**) Transfer curves measured at different temperatures for transistor with 2-nm AlN/18-nm Al_2_O_3_ gate dielectric. The sweep time is 70 s and the drain bias is 0.1 V. Insets show the zoomed-in transfer curves during the up- and down-sweep. (**b**) Hysteresis extracted from (**a**).

It has been demonstrated that MoS_2_ transistor is capable of working at high temperature, although significant mobility degradation and threshold voltage shift have been observed^46^. The MoS_2_ transistors fabricated in this work with 2-nm AlN/18-nm Al_2_O_3_ gate dielectric was tested at temperatures ranging from 25 °C to 100 °C, all conducted in ambient environment. The transfer curves are plotted in Fig. 5a, with a sweep time of 70 s. The two insets of Fig. 5a show the zoomed-in transfer curves, from which we can clearly see the shifting trends of the transfer curves with increased temperature. A negative shift in *V*_th_ is observed at higher temperature, which is caused by enhanced emission of electrons trapped in the gate dielectric. The hysteresis increases at higher temperature, as quantitatively shown in Fig. 5b. This is because the gate leakage and the consequent electron injection/trapping is enhanced at higher temperature. Nevertheless, the hysteresis still remains smaller than 0.5 V.

**Figure 6:**
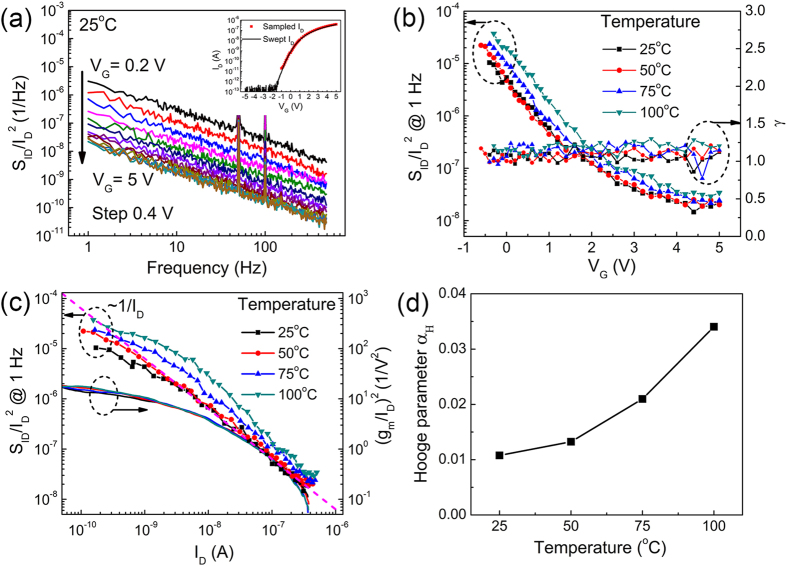
(**a**) Normalized current power spectra 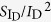 for different gate biases at 25 °C for transistor with 2-nm AlN/18-nm Al_2_O_3_ gate dielectric. The inset shows the average sampled *I*_D_ (red) during the power spectrum measurement in comparison with the swept transfer curve (black). (**b**) 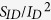 at 1 Hz and fitted frequency exponent γ for different temperatures. (**c**) Plots of 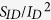 at 1 Hz and (*g*_m_/*I*_D_)^2^ versus *I*_D_. (**d**) Hooge parameter *α*_H_ extracted from (**c**).

Nanoscale devices can suffer from the flicker noise (1/f noise) which increases with smaller channel size^47^. Thus, maintaining a low level of flicker noise is necessary to obtain high performance MoS_2_ transistors. On the other hand, the flicker noise characterization is a useful tool to diagnose the channel/gate-dielectric interface^20^,^47^,^48^, since the conventional C-V measurement has become difficult due to the limited channel area. The flicker noise spectra for the fabricated MoS_2_ transistor with 2-nm AlN/18-nm Al_2_O_3_ gate dielectric at various temperatures were measured using SR570 low-noise current preamplifier. The results at 25 °C are shown in Fig. 6a. The spectra correspond to different gate biases with a fixed drain bias of 0.1 V. The measurement of each spectrum takes a total sampling time of 120 s. The inset of Fig. 6a shows the average sampled *I*_D_ (red), together with the quasi-static *I*_D_ (black), showing no obvious discrepancy. This agreement suggests again very good electrical stability of the MoS_2_ transistors even after long-term electrical stress. The normalized flicker noise can be expressed as 
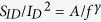
, in which *A* is the noise amplitude and *f* is the frequency. Ideally the frequency exponent *γ* should be close to 1^20^. Figure 6b shows the noise amplitude *A* (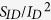 at 1 Hz) and also the fitted frequency exponent *γ*, for different gate biases at various temperatures. All the values of *γ* are close to 1, suggesting that the noise spectra follow the 1/*f* relationship well. Meanwhile, we find that higher flicker noise occur at higher temperatures. The normalized noise 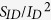 at 1 Hz decrease monotonously with the gate voltage, suggesting that there is less relative fluctuation when more carriers are present in the channel.

The generation of flicker noise can be explained by the carrier number fluctuation or the mobility fluctuation^49^. For noise generated by carrier number fluctuation, the flicker noise 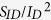 is proportional to (*g*_*m*_/*I*_*D*_)^2^, where *g*_*m*_ is the gate transconductance, while the flicker noise 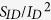 generated by the mobility fluctuation would be simply proportional to 1/*I*_*D*_^49^. To find the exact origin of the flicker noise, 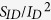 (at 1 Hz) and (*g*_*m*_/*I*_*D*_)^2^ are plotted together in Fig. 6c. We find that 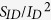 deviates from (*g*_*m*_/*I*_*D*_)^2^ significantly by several orders. On the other hand, 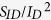 follows 1/*I*_*D*_ relationship well as indicated by the dashed line. Therefore, we conclude that the flicker noise observed in our MoS_2_ transistor is caused by the mobility fluctuation instead of the carrier number fluctuation, which is consistent with previous report for single-layer MoS_2_ transistor^20^.

According to the Hooge empirical relationship, the flicker noise can be expressed by 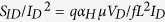
47. The Hooge parameter *α*_H_ is extracted and plotted in Fig. 6d. It can be seen that the Hooge parameter increases with temperature, indicating severer mobility fluctuation at higher temperature. The measured Hooge parameter at 25 °C is 0.011, which is two orders of magnitude smaller than the published result for the same mobility range^20^, suggesting excellent suppression of flicker noise. The flicker noise caused by carrier number fluctuation is related to the border trap density by equation 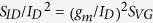, in which 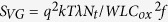 is the equivalent gate voltage spectral density^47^. Since in our case the flicker noise is generated by the mobility fluctuation, it is difficult to extract the accurate border trap density directly, but the observed flicker noise has placed an upper limit on the possible border trap density. By assuming the flicker noise is all generated by the carrier number fluctuation when *V*_G_ is biased above the threshold voltage at 25 °C, we calculate the upper limit of the border trap density to be *λN*_*t*_ ≪ 1.6 × 10^12^*cm*^−2^*eV*^−1^ (See Supplementary Figure S6 for the results of *S*_*VG*_).

## Conclusion

Single-layer MoS_2_ MOSFETs with AlN/Al_2_O_3_ gate dielectric are demonstrated. Gate dielectrics scaling down to 6 nm on the dangling-bond free MoS_2_ are realized by the insertion of AlN interfacial layer. The MoS_2_ transistor with 20-nm top-gate dielectric exhibits hysteresis smaller than 0.1 V at room temperature in ambient environment. The hysteresis increases when biased at higher gate voltage or measured at higher temperature, but remains below 0.8 V even for gate-dielectric electric-field as high as 4.5 MV/cm or temperature up to 100 °C. This remarkable electrical stability mainly benefits from the low border trap density, enabled by the inserted AlN interfacial layer, and consequently small gate leakage and high dielectric strength of the AlN/Al_2_O_3_ stack. The MoS_2_ MOSFET also presents a low level of flicker noise, which is generated by the mobility fluctuation instead of the carrier number fluctuation. AlN/Al_2_O_3_ with AlN as interfacial layer is shown to be a promising candidate as both excellent gate dielectric and effective passivation for implementing reliable MoS_2_ MOSFETs, and probably also has the potential to be used in other nanodevices, such as transistors based on graphene, carbon nanotubes and other TMDs, which also face the problems of large hysteresis and reliable dielectric deposition due to the lack of surface bond.

## Methods

### MoS_2_ Preparation

MoS_2_ flakes were synthesized on sapphire substrate by CVD method using high purity MoO_3_ and S powder as precursors. The synthesized MoS_2_ has a flake size of about 100 μm, and the layer thickness was checked by Raman measurement (514 nm, inVia Renishaw). The Raman characterization shows peak distance of 19 cm^−1^ for 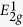 and *A*_1*g*_, suggesting the MoS_2_ flakes are single layered. To transfer the MoS_2_ flakes to Si substrate covered by 300 nm thermal SiO_2_, the sample was first spin coated with PMMA A4 at 3000 rpm and baked at 130 °C for 2 min, then soaked in 10% KOH at 80 °C for hours until the PMMA membrane was separated from the sapphire substrate and floated on the water surface. The PMMA membrane was fished to DIW for several times and finally fished to the Si target substrate.

### Transistor Fabrication

Source/drain contacts were first formed by EBL (Raith e-line), followed by e-beam evaporation of 10-nm Ti and 50-nm Au and lift-off in acetone. MoS_2_ flakes were then patterned to the desired channel size by EBL and O_2_ plasma etch. Both AlN and Al_2_O_3_ were deposited using the Oxford Instruments OpAL ALD system. AlN was deposited at 170 °C by using trimethylaluminum (TMA) and remote N_2_ plasma (20 sccm with a coil power of 25 W) as Al and N sources. The growth temperature and the RF power were optimized to minimize the plasma damage to the MoS_2_, resulting in a relatively slow growth rate of 0.18 Å/cycle. After the deposition of AlN, Al_2_O_3_ was grown *in-situ* under thermal ALD mode at 200 °C by using TMA and water vapor as Al and O sources. Two kinds of AlN/Al_2_O_3_ dielectric stacks consisting of 2-nm AlN/18-nm Al_2_O_3_ or 1-nm AlN/5-nm Al_2_O_3_ are deposited. The thicknesses of AlN and Al_2_O_3_ were verified by ellipsometer on the Si dummy wafer (J.A. Woollam M-2000V). During the measurement, 1.5 nm native SiO_2_ layer was taken into consideration. A Cauchy dielectric model for AlN was developed by fitting the Ellipsometer data measured from 18.5 nm AlN. Another EBL was conducted to define the 3-μm-long gate electrode made of Ti/Au (10 nm/50 nm). Finally the source and drain contact holes were formed by EBL and wet etch in FHD-5 for 10 min. After the fabrication, no further annealing was conducted.

### Characterization

The successful deposition of AlN and Al_2_O_3_ thin films was verified by TOF-SIMS V (ION-TOF GmbH, Münster, Germany), with an analysis spot size of 41 μm × 41 μm. The surface of MoS_2_ after deposition was checked by AFM (XE150S, Park system). The cross-sectional view of MoS_2_/AlN/Al_2_O_3_ was done by TEM (JEOL 2010F). The electric device performances were measured by Agilent B1505A device analyzer/curve tracer, inside a probe station equipped with a thermal chuck. To measure the low frequency noise, the transistor was first biased at a fixed gate voltage and drain voltage (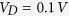), then the drain current was amplified by the low noise current preamplifier (SR570, Stanford Research System) and sampled for a total time of 120 s. Later on Fourier transformation was performed to the recorded current for every 4 s, and the final single-sided current power spectrum was obtained by averaging the 30 independent results.

## Additional Information

**How to cite this article**: Qian, Q. *et al.* Improved Gate Dielectric Deposition and Enhanced Electrical Stability for Single-Layer MoS_2_ MOSFET with an AlN Interfacial Layer. *Sci. Rep.* **6**, 27676; doi: 10.1038/srep27676 (2016).

## Supplementary Material

Supplementary Information
