# Supplementary material for: Improved Gate Dielectric Deposition and Enhanced Electrical Stability for Single-Layer MoS2 MOSFET with an AlN Interfacial Layer
Source: Sci Rep. 2016 Jun 9;6:27676. doi: 10.1038/srep27676 (PMC4899804; doi:10.1038/srep27676)
Supplement: Supplementary Information [file srep27676-s1.pdf]

Supplementary Information

Improved Gate Dielectric Deposition and Enhanced Electrical Stability for Single-Layer MoS_2 MOSFET with an AlN Interfacial Layer

Qingkai Qian,[1] Baikui Li,[1] Mengyuan Hua,[1] Zhaofu Zhang,[1] Feifei Lan,[2] Yongkuan Xu,[2] Ruyue Yan,[2] and Kevin J. Chen[1,]*

[1]Department of Electronic and Computer Engineering, Hong Kong University of Science and Technology, Clear Water Bay, Hong Kong SAR, China.

[2]The 46th Research Institute, CETC, Tianjin 300220, China.

Correspondence and requests for materials should be addressed to K.J.C. (email: eekjchen@ust.hk)

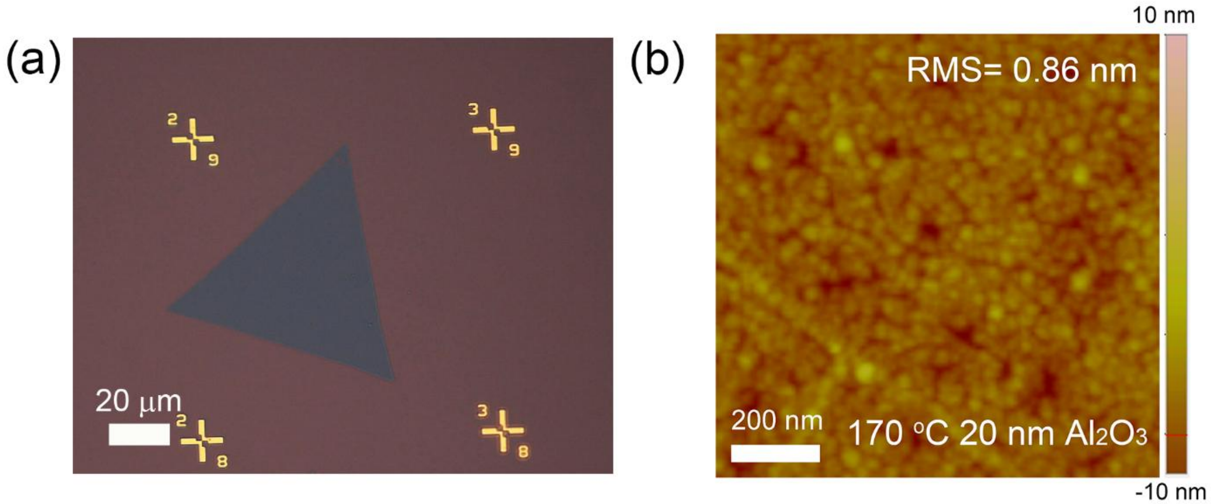

Supplementary Figure S1 | (a) Microscopic image of single-layer MoS_2 flake after being transferred to Si substrate capped with 300 nm SiO_2. (b) AFM image of MoS_2 surface after the direct deposition of 20-nm Al_2O_3 at 170 °C. Pinholes are still observed even though with a lower growth temperature.

AlN was deposited by using trimethylaluminum (TMA) and remote N_2 plasma (with controllable N_2/H_2/Ar composition and adjustable RF coil power) as Al and N sources respectively. During one cycle of AlN growth, 40 ms TMA with carrier gas of 10 sccm Ar is provided for the Al source first, then the chamber is purged by 30 sccm Ar for 3 s. Considering that only 20 ms TMA dosage was used for ALD Al_2O_3 and had already resulted in a growth rate of 1 Å/cycle, 40 ms TMA for ALD AlN can be regarded as over dosed. 60 s and 20 sccm N_2 plasma with an adjustable Ar/H_2 content and remote RF coil power is used as N source.

Supplementary Figure S2(a) shows the Raman signal of single-layer MoS_2 after the deposition of AlN with different RF powers for 80 cycles. Higher power causes a weaker Raman signal, suggesting more defects generated. As a trade-off of growth rate and plasma damage, 25 W is adopted. We find that the deposition of AlN with pure N_2 at 150 °C as N source results in particles

as shown in Supplementary Figure S2(b), meaning gas-phase reaction happens. By adding a small amount of 5 sccm Ar or 5 sccm H_2 together with 20 sccm N_2 as the N source, particles can be eliminated. However as shown in Supplementary Figure S2(c), the Raman signal decreases a lot, suggesting compared to the mild N_2 plasma, the more reactive H or heavier Ar atom causes more defects to the fragile MoS_2[1]. Increasing the growth temperature is found to be an alternative way to eliminate particles and at the same time to mitigate the plasma damage to MoS_2 as shown in Supplementary Figure S2(d).

The growth rate of ALD AlN with different temperatures and RF powers are summarized in Supplementary Figure S3. The growth rate decreases slightly with increasing temperature, which is due to the enhanced thermal desorption of the precursor from the substrate.[2,3] With high enough power, the growth rate gradually begins to saturate at about 1 Å/cycle, which is close to other reported result and is sign of ALD growth.[3]

Based on the above observation, 25 W remote RF plasma power, 20sccm pure N_2 as N source and growth temperature of 170 °C are used as the growth condition for AlN. Even though the growth temperature is not in the ideal ALD window, and the growth rate is not saturated yet due to the small RF power, the reason to use these parameters is to minimize the plasma damage and to demonstrate the potential of AlN interfacial layer as much as possible, even for single-layer MoS_2. Compare to the fresh MoS_2 before ALD AlN, the contrast experiment in Supplementary Figure S4 suggests that the remaining damage is probably caused by the diffusion and activation of Ar (used as both carrier and purge gas of TMA) or H (from decomposition of TMA) to the remote RF coil. By further optimization of the carrier and purge gas for TMA, the plasma damage can be greatly reduced even for single-layer MoS_2.

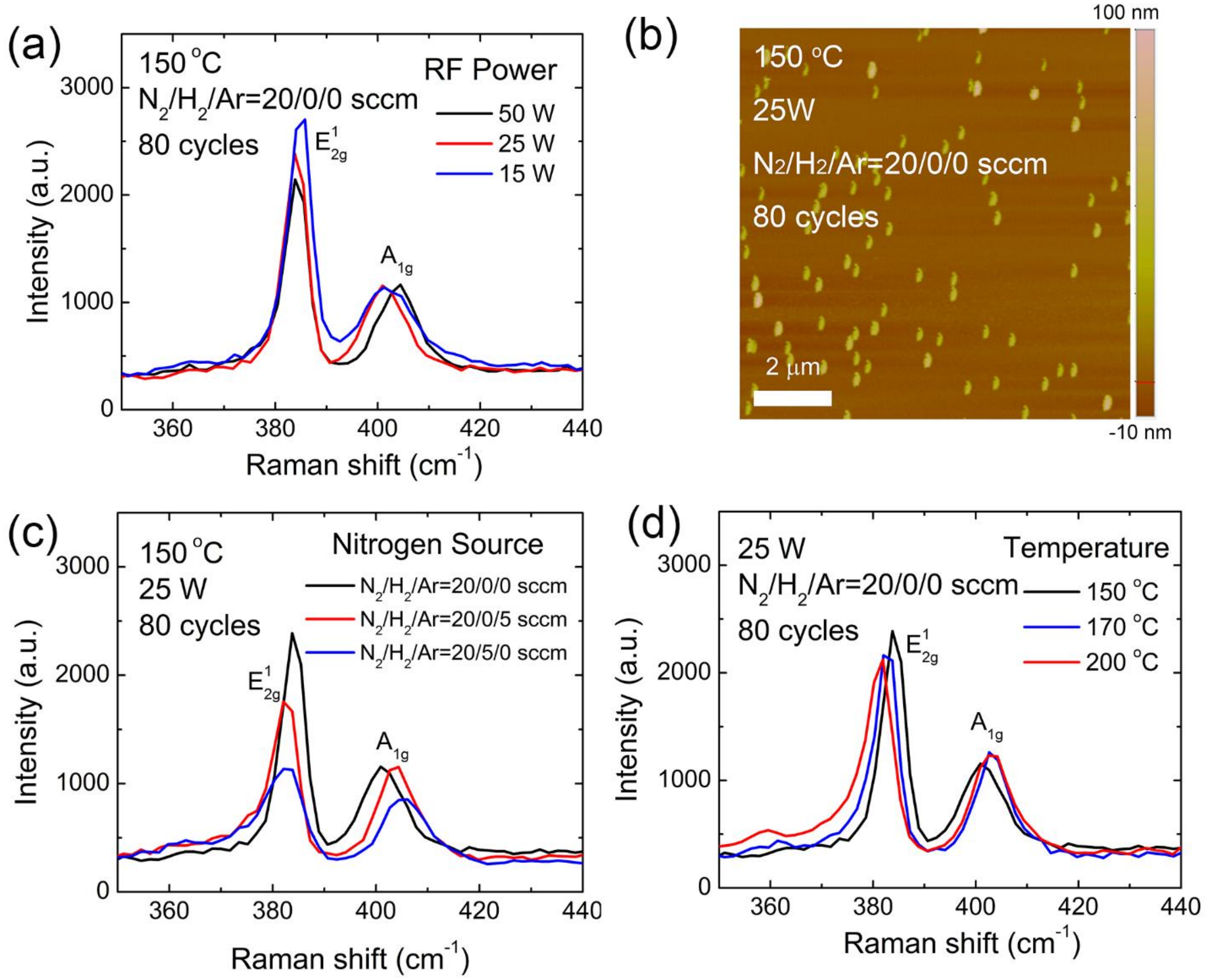

Supplementary Figure S2 | Deposition condition optimization for AlN growth. Different remote RF powers, gas compositions as nitrogen source and deposition temperatures are considered. (a) Raman spectra of single-layer MoS_2 after AlN deposition with different RF powers. The growth temperature is 150 °C. Pure 20 sccm N_2 is used as the nitrogen source. (b) AFM image of MoS_2 surface after AlN deposition at 150 °C with RF power of 25 W. Particles are generated, meaning gas phase reaction happens. (c) Raman spectra of MoS_2 after AlN deposition with different gas compositions as the nitrogen source, by adding 5 sccm Ar or 5 sccm H_2, particles are avoided, but Raman signal decreases a lot. (d) Raman spectra of MoS_2 after AlN deposition at

different temperatures. With temperatures higher than 170 °C, particles are eliminated, and at the same time the AlN growth only causes a relatively mild damage.

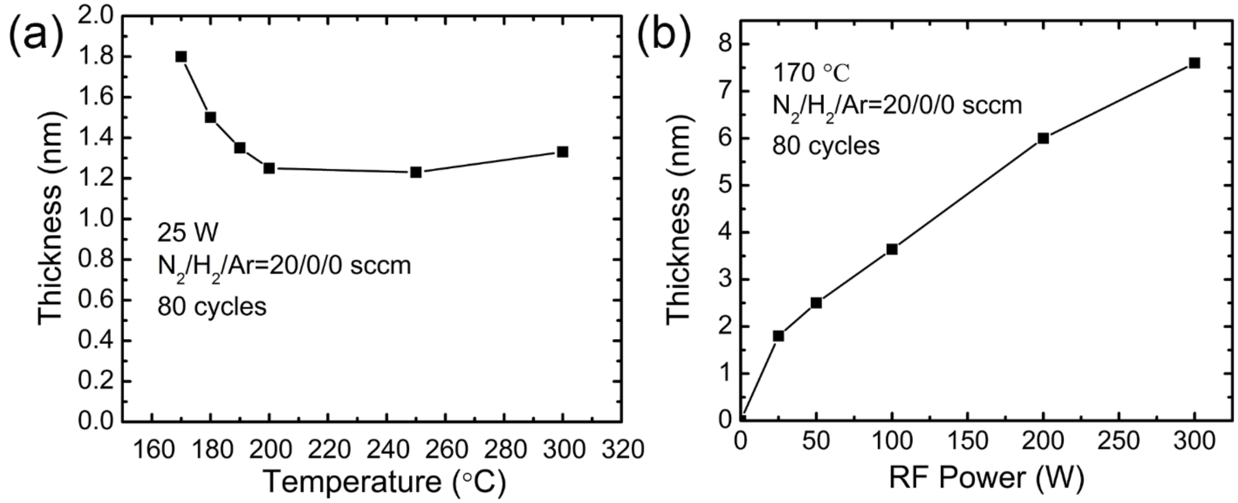

Supplementary Figure S3 | ALD growth rate of AlN for different temperatures and RF powers. (a) ALD growth rate as a function of temperature. ALD growth rate decreases slightly with higher temperature due to increased thermal desorption of TMA precursor[2,3]. (b) ALD growth rate as a function of RF power. With high RF power, the growth rate begins to saturate.

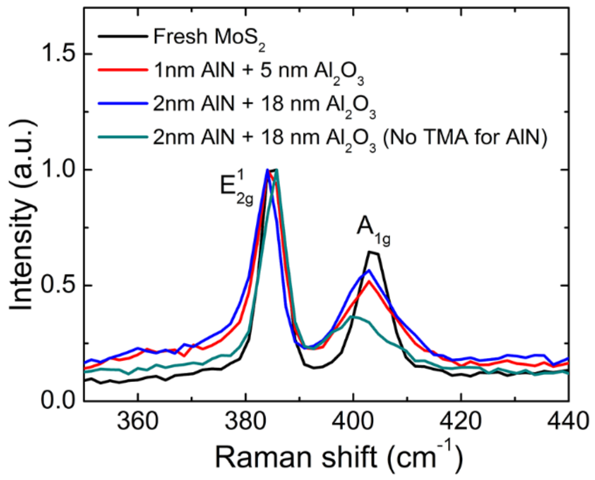

Supplementary Figure S4 | Raman spectra of MoS_2 after AlN/Al_2O_3 deposition and contrast experiment to identify the origin of plasma damage. Raman signals are normalized by E_{2g}^1

peaks. The defects can be judged by the peak width of E^1_{2g}, because it is less sensitive to the doping influence compared to A_{1g}[4]. After AlN/Al_2O_3 deposition, Raman peak E^1_{2g} broadens, indicating defects generated during the PEALD of AlN[5]. However the contrast experiment of cutting off TMA source and only providing pure N_2 plasma for AlN growth (dark cyan) results in almost no defects, suggesting that the defects are not induced by the N_2 plasma but probably caused by the diffusion of Ar/H (Ar is used to carry and purge out TMA, H comes from decomposition of TMA) to the remote RF coil, which becomes activated and causes similar damage to MoS_2 as Supplementary Figure S2(c).

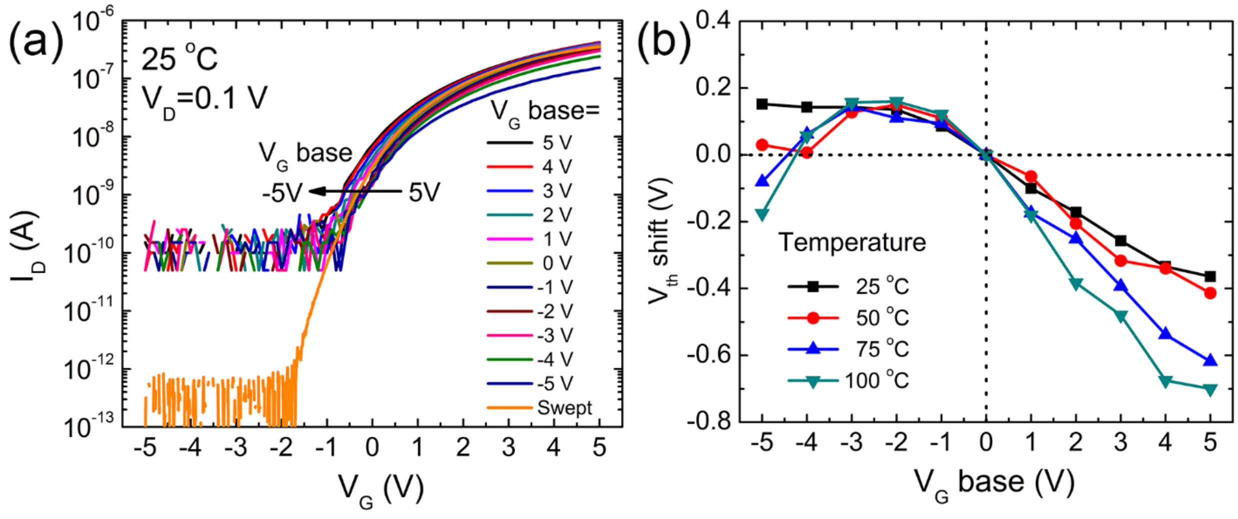

Supplementary Figure S5 | (a) Pulsed I-V measurement of transfer curves with different V_G base at 25 °C. The pulse width is 2 ms. With V_G base decreasing from 5 V to -5 V, the V_{th} shifts to the left slightly, indicating mainly gate side injection. The yellow line is the normally swept transfer curve. (b) V_{th} shift for different V_G base. The V_{th} is extracted by criterion of $I_D = 10^{-9}A$, and results for different temperatures are measured. With higher temperature, the positive bias causes more gate side injection, but the negative bias has started to show more MoS_2 channel side injection.

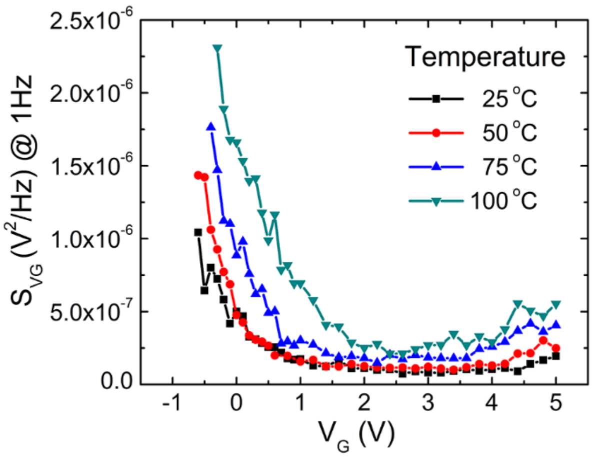

Supplementary Figure S6 | The equivalent gate voltage spectral density S_{VG} as a function of different gate biases and temperatures. The drastic increases of S_{VG} at biases below the threshold voltage are also signs of Hooge mobility fluctuation[6].

References

1. Kim, B. H. et al. Effect of sulphur vacancy on geometric and electronic structure of MoS2 induced by molecular hydrogen treatment at room temperature. *RSC Adv.* **3,** 18424 (2013).
2. Koo, J. et al. Characteristics of Al_2O_3 Thin Films Deposited Using Dimethylaluminum Isopropoxide and Trimethylaluminum Precursors by the Plasma-Enhanced Atomic-Layer Deposition Method. *J. Korean Phys. Soc.* **48,** 131-136 (2006).
3. Kim, K., Kwak, N. & Lee, S. H. Fabrication and Properties of AlN Film on GaN Substrate by Using Remote Plasma Atomic Layer Deposition Method. *Electron. Mater. Lett.* **5,** 83-86 (2009).
4. Chakraborty, B. et al. Symmetry-dependent phonon renormalization in monolayer MoS2 transistor. *Phys. Rev. B* **85,** (2012).
5. Mignuzzi, S. et al. Effect of disorder on Raman scattering of single-layer MoS2. *Phys. Rev. B* **91,** (2015).
6. Ghibaudo, G., Roux, O., Nguyenduc, C., Balestra, F. & Brini, J. Improved Analysis of Low Frequency Noise in Field-Effect MOS Transistors. *Phys. Status Solidi A* **124,** 571-581 (1991).
